# Perceived progress toward scale of 14 maternal, newborn, child health, and nutrition (MNCHN) assets at subnational level in Burkina Faso, Ethiopia, India, Kenya, and Nigeria

**DOI:** 10.1371/journal.pgph.0002309

**Published:** 2024-03-15

**Authors:** Patricia S. Coffey, Sadaf Khan, Elan Ebeling, Cyril Engmann

**Affiliations:** PATH, Seattle, Washington, United States of America; PLOS: Public Library of Science, UNITED STATES

## Abstract

Inequitable coverage of evidence-based MNCHN interventions is particularly pronounced in low and middle income countries where access and delivery of these interventions can vary dramatically at the subnational level. We conducted health system assessments in nine subnational geographies in five countries (Burkina Faso, Ethiopia, India, Kenya and Nigeria) to explore progress toward scale of 14 evidence-based MNCHN interventions (iron-folic acid, oxytocin, magnesium sulfate, misoprostol; 7.1% chlorhexidine for umbilical cord care, neonatal resuscitation, kangaroo mother care, community regimen for the treatment of possible severe bacterial infection; amoxicillin dispersible tablets, multiple micronutrient supplements, balanced energy protein supplementation, early and exclusive breastfeeding, feeding of small and sick newborns, and management of severe and moderate acute malnutrition in children less than five years old). Between March and October 2021, we conducted key informant interviews with a purposive sample of 275 healthcare providers and 94 district health management (DHMT) staff to better understand bottlenecks, facilitators and uptake of the interventions across varied subnational settings. Across all interventions and geographies, providers and DHMT staff perceived lack of robust HMIS data as the most significant barrier to scale followed by weak facility infrastructure. DHMT staff viewed limited budget allocation and training as a much larger barrier than healthcare providers, most likely given their purview as subnational managers. Healthcare providers were focused on supply chain and staffing, which affect workflows and service provision. Understanding provider and health facility management views of why interventions do or do not advance towards effective coverage can assist in creating enabling environments for the scale of best practices. These types of data are most helpful when collected at the subnational level, which allows for comparisons both within and between countries to show health disparities. Importantly, this strategic data collection can provide a starting point for improvement efforts to address existing health system gaps.

## Introduction

Scale-up and effective coverage of evidence-based reproductive maternal, newborn, child health, and nutrition (RMNCHN) practices is a high-yield, deeply effective, and smart investment to make. Besides the obvious moral imperative, the health and economic return on investment makes a compelling case for the same. Modeling of health and socioeconomic returns of investments in health systems strengthening suggest reduction in preventable maternal, newborn, and child death, as well as gains in gross domestic product and productivity [1]. Scaling of ten evidence-based maternal and child nutrition interventions at 90% coverage could result in a 15% reduction in death of children under 5 years [2]. A 2012 United Nations Commission for Lifesaving Commodities review estimated that for an investment of $2.6 billion, six million lives could have been saved between 2010 and 2015, if 13 lifesaving commodities were scaled and effective coverage attained [3]. Following this review, a newly established RMNCH fund committed US$175.7 million to 19 countries to support strategies to advance the 13 lifesaving commodities with about forty percent of these funds going towards support of health systems strengthening (provider training, supply chain, and demand generation) [4]. Yet a decade later, gaps in effective coverage of key evidence-based interventions persist [5].

Recent analyses from 2020 conclude that scaling up essential RMNCH interventions to reduce gaps in inequitable coverage is critical to achieving the Sustainable Development Goals in LMICs by 2030 [6]. There are numerous challenges to ensuring equitable scale-up and coverage of evidence-based interventions. These challenges are especially pronounced in southeast Asia and Sub-Saharan Africa, where delivery can vary dramatically. There is also a relative paucity of real-world data that analyzes the effective coverage of evidence-based RMNCHN interventions.

Inequitable coverage of evidence-based RMNCH interventions is particularly pronounced in sub-Saharan Africa where delivery of these interventions can vary dramatically at the subnational level [7, 8]. To address these gaps and advance understanding of the numerous challenges to scaling effective coverage, we assessed the programmatic implementation of 14 evidence-based RMNCHN practices (called “assets” for the purposes of this assessment). Maternal assets included iron-folic acid, oxytocin, magnesium sulfate, and misoprostol; newborn assets included 7.1% chlorhexidine for umbilical cord care, neonatal resuscitation, kangaroo mother care, and community regimen for the treatment of possible severe bacterial infection (PSBI); child assets included amoxicillin dispersible tablets; nutrition assets included multiple micronutrient supplements, balanced energy protein supplementation, early and exclusive breastfeeding, feeding of small and sick newborns, and management of severe and moderate acute malnutrition in children less than five years old. This assessment was conducted at the subnational level in five key geographies. This effort stemmed from an earlier investigation of the national-level coverage and impact of nine assets across seven countries (Burkina Faso, Ethiopia, India, Kenya, Malawi, Nigeria, and Tanzania) [9]. In the current assessment, we focused on availability, service delivery, use, and community-level demand and access at the subnational level only. The objectives of the assessment were to:

1. Understand the level of awareness about the assets among different stakeholders.
2. Assess the level of policy operationalization, implementation, and delivery associated with these assets.
3. Understand the bottlenecks and facilitators in implementation and uptake of the assets across different geographical regions.
4. Ascertain the availability of drugs/devices/supplements/tools associated with the 14 assets at the facility/community health. [reported elsewhere]

## Methods

This exploratory, mixed-methods assessment at subnational level explored the progress towards scale of 14 maternal, newborn, child health, and nutrition interventions or “assets.”

**Fig 1:**
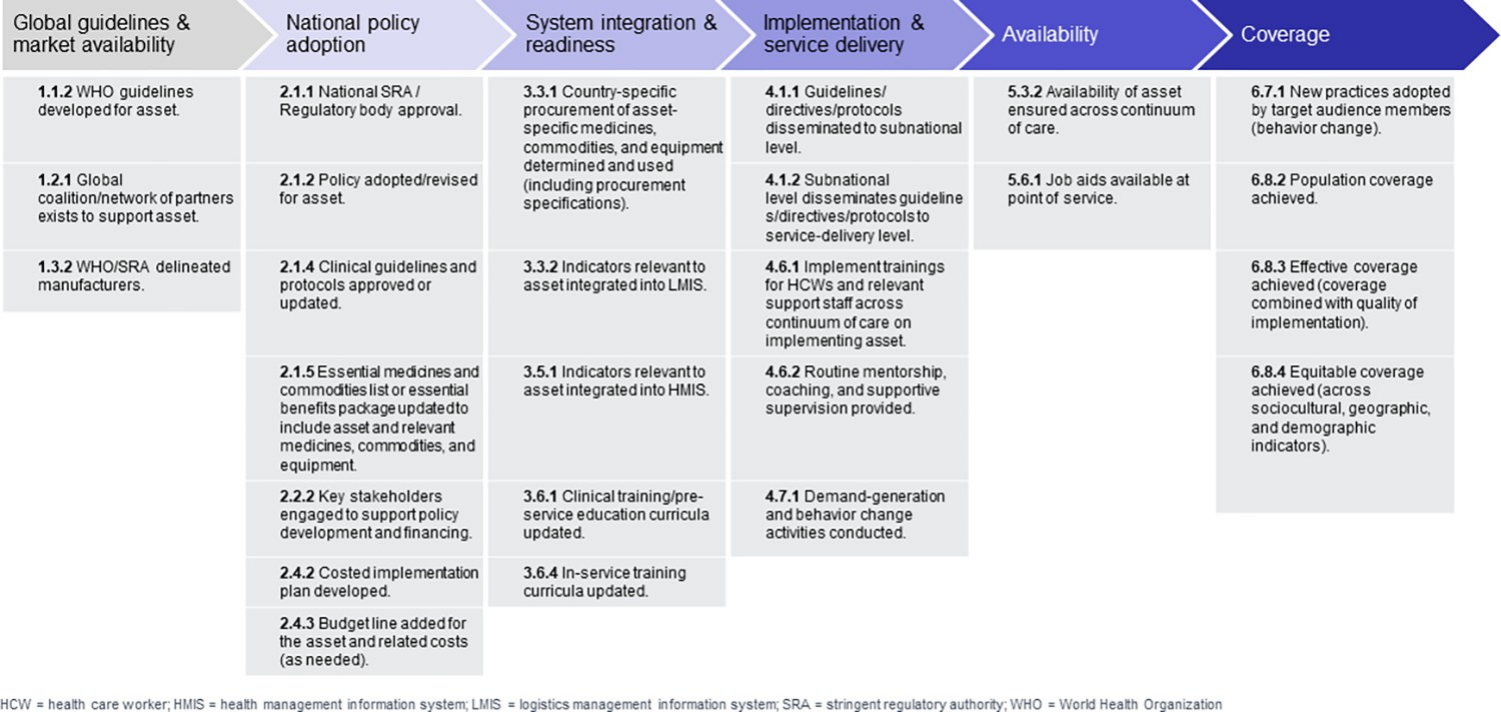
Six stage framework of the pathway to effective coverage. HCW = health care worker; HMIS = health management information system; LMIS = logistics management information system; SRA = stringent regulatory authority; WHO = World Health Organization.

Based on a review of implementation science frameworks [10–14] in 2019, we developed a six-stage framework (Fig 1) to codify phases of scale-up, which serves as the overarching framework for organizing the data collection and analysis of this study as well as development of interactive dashboards to visualize the status of assets along the pathway toward effective coverage.

This framework conceptualizes the typical pathway assets take as they move toward scale. We identified key milestones that assets typically achieve on the pathway to scale (access, uptake, implementation, and coverage), and identified available indicators and data to measure the milestones. Previous investigation at the national level [15] found that some milestone indicators are verifiable across a variety of data sources, while other milestone indicators had limited or no data availability in policy documents, existing surveys, country databases, or other routine sources. This is particularly relevant for indicators related to “system integration and readiness” and “implementation and service delivery”, which require subnational level measurements.

The 14 assets selected by the funding organization are described in Table 1. For interventions with multiple clinical indications (e.g oxytocin, misoprostol) these definitions also delineated the scope which would be utilized in this inquiry.

**Table 1:** MNCHN assets of interest.

Asset	Description
**Iron Folic Acid**	Daily oral iron and folic acid supplementation for pregnant women with 30 mg to 60 mg of elemental iron* and 400 μg (0.4 mg) folic acid; oral supplements are available as capsules or tablets (including soluble tablets, and dissolvable and modified-release tablets).
(Maternal health)	
**Oxytocin**	10 IU oxytocin (IM/IV) use as part of active management for the third stage of labor (AMTSL) for post-partum hemorrhage (PPH) prevention and for management of PPH.
(Maternal health)	
**Magnesium Sulfate**	Magnesium sulfate is recommended for the prevention of eclampsia in women with severe pre-eclampsia and the treatment of women with eclampsia. It is administered by intravenous (IV) and intramuscular (IM) routes.
(Maternal health)	
**Misoprostol**	Oral misoprostol for prevention and treatment of PPH by health workers trained in its use (or as indicated by country guidelines).
(Maternal health)	
**7.1% Chlorhexidine for umbilical cord care**	7.1% chlorhexidine digluconate, which delivers 4% chlorhexidine applied as single dose or once daily for 7–10 days or until cord falls off for umbilical cord care; the product comes in 3 formulations: gel, liquid, and impregnated sachet.
(Newborn health)	
**Newborn resuscitation**	The practice and group of commodities needed to provide and maintain the skills in basic neonatal resuscitation (neonatal self-inflating bag; masks size 0 and 1; reusable manual suction device; training mannequin; Helping Babies Breathe educational materials or equivalent).
(Newborn health)	
**Kangaroo Mother Care**	Prolonged skin-to-skin contact between mother and infant, exclusive breastfeeding whenever possible, early discharge with adequate follow-up and support, and initiation of the practice in the facility and continuation at home for the management of low-birthweight newborns (<2000 g at birth).
(Newborn health)	
**Community Regimen for the Treatment of Possible Severe Bacterial Infection (PSBI)**	The implementation of a WHO guideline that recommends simplified antibiotic treatment for possible serious bacterial infection in sick young infants aged 0–59 days at outpatient primary care level when referral is not feasible.
(Newborn health)	
**Amoxicillin Dispersible Tablet**(Child health)	Amoxicillin 250mg Dispersible Tablet for pneumonia among children between the age of two months and five years old in community and primary care settings.
**Multiple micronutrient supplements** (Nutrition)	Daily multiple micronutrient supplement for pregnant women containing 13 to 15 micronutrients (including iron and folic acid); oral supplements obtained by pregnant women receiving antenatal care in any health care facility or community-based setting and/or purchased from local chemists.
**Balanced energy protein supplementation**	Delivery of balanced energy protein supplementation products (macronutrient food‐based supplements where the protein provides less than 25% of the total energy content that can be in the form of biscuits, milk, or both/other food products) during pregnancy for women with dietary deficiencies, aged15 to 49 years.
(Nutrition)	
**Early and Exclusive Breastfeeding**	Optimal support for mothers to initiate breastfeeding as soon as possible after birth, within the first hour after delivery and, for exclusive breastfeeding, defined as no other food or drink—not even water—except breast milk (including milk expressed or from a wet nurse) for six months of life, and allows the infant to receive oral rehydration solution, drops and syrups (vitamins, minerals, and medicines).
(Nutrition)	
**Feeding of small and sick newborns**	Small and sick newborns receive human milk (mother’s own milk, donor human milk), fortified when necessary, to meet their unique nutritional needs; alternative feeding is available when needed (donor human milk or formula).
(Nutrition)	
**Management of severe and moderate acute malnutrition in children less than five years old**	Approach to screen, diagnose, and treat cases of severe and moderate acute malnutrition in children less than five including 0–6 months.
(Nutrition)	

### Country selection

**Fig 2:**
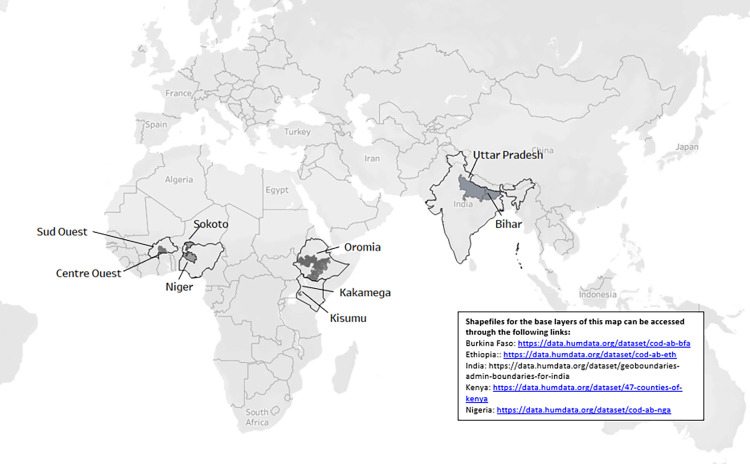
Map of selected subnational geographies within countries.

Country selection was based on funder request. We conducted subnational health system assessments using mixed methods in five countries. In four of the countries, two subnational geographies were represented: Burkina Faso (Centre Ouest and Sud Ouest regions), India (Uttar Pradesh and Bihar states), Kenya (Kakamega and Kisumu counties), and Nigeria (Niger and Sokoto states). In Ethiopia, this work was conducted only in the Oromia region (Fig 2).

### Health district and health facility selection and sampling strategy

In each country we tailored our health district and facility selection strategy, based on contextual needs. We worked closely with the Ministry of Health (MOH) at both national and subnational levels to agree on the selection criteria and the possible geographies within each subnational unit. Site selection was based on the health care structure, staffing of facilities, and consultations with local stakeholders, including subnational authorities.

Within each subnational unit, we purposively selected 4–8 health district administrative areas using the following criteria:

- Level of performance at district level vis-à-vis morbidity, mortality, and uptake of MNCH services.
- Heterogeneity of subunits vis-à-vis economic and socio-demographic variables.
- Emergency obstetric care, essential newborn care services, and services for small and sick newborns available in at least one facility within the subunit.
- Willingness of district-level authorities to allow data collection at all facilities within the district and safe access and travel to and within the district.
- To the extent possible we tried to capture geographic spread within the district.

Criteria for facility selection were expansive to account for health facilities of all sizes. An effort was made to include peripheral and smaller health facilities and birthing homes. We excluded facilities not staffed by providers meeting the local definition of skilled birth attendants. To ensure representation from a large hospital in each area, we included one comprehensive emergency obstetric and newborn care facility in each subnational region.

### Stakeholder selection

We used purposive sampling to identify respondents who were skilled staff at facility level (i.e., medical officer/physician, pediatrician, obstetrician/gynecologist, nurse, midwife, nutritionist/dietician, facility manager/administrator such as health medical record staff, pharmacist, and laboratory technician). For each subnational unit, we interviewed managers of district health management staff or the local equivalent. Given the exploratory nature of this work, no sample size calculations were done. The number of participants was based on logistical and budget limitations.

Eligibility criteria to participate in this assessment included:

- 18 years of age or older
- Be a (i) member of the district management team, (ii) facility manager, (iii) provider currently offering services at one of the selected health facilities involved in provision of MNCHN services, or (iv) community health worker currently working in the catchment area of the selected facility who is the mother of at least one child five years of age or younger
- Agree to be audio-recorded during any discussion or interview

Exclusion criteria are noted below:

- Younger than 18 years of age
- Community health workers who are not mothers of children aged five years or less
- Not currently providing a health care service at the facility or community level
- Eligible participants who did not consent were automatically excluded from this assessment

### Data collection and management

Data collection utilized a single sample strategy and a mixed-methods design including observations and interviews. Primary data collection consisted of key informant interviews with district health management teams and providers at health facilities, discussions with community health workers (not reported here), and spot checks to ascertain the availability of drugs/devices/supplements associated with the 14 assets at facility/community health level (not reported here).

At each health facility two to five key informant interviews were conducted based on the level of the facility, to include representatives of each provider category and until saturation was reached. The semi-structured questionnaire explored their perceived role in management/care provision, typical workflow, decision-making around health service provision, stocking process, record-keeping, supply chain, asset availability and uptake, stock-out frequency and inventory order process, facility infrastructure challenges, and constraints and barriers to effective care of mothers and neonates with a focus on the 14 assets. Quantitative data pertained to respondent level of familiarity and use of the fourteen assets, if they considered any of the five barrier domains (policy, data management, staffing, infrastructure, budget) to be a barrier to scale generally and specifically for each asset. We also asked respondents their perspective on how well or poorly each asset had scaled using a ten-point Likert-type scale. For each asset, we asked respondents to verify the existence of a relevant national policy and/or subnational care guideline and assessed the status of nine milestone indicators that aligned with the six-stage pathway to effective coverage model. The milestone indicators were asset-specific and queried yes/no responses about dissemination of subnational guideline to facilities, conduct of training, routine mentorship, coaching and supportive supervision, availability of job aids, commodity and funds for procurement, data entry and use and demand generation.

All data collection instruments were translated into the appropriate local language. The priority for translation was to create equivalent meaning rather than literal translation to ensure that the data collected were valid and comparable. Data were collected via face-to-face through in-facility interviews using CAPI (Computer Assisted Personal Interview) on android tablet and/or mobile phone with adherence to guidelines and protocols developed because of the COVID-19 pandemic. The CAPI data collection system included logical checks and balances based on the survey tool (in-built condition), a cascading-style of questions based on an appropriate skip pattern to ensure a smooth flow of questions and coherence during administration and a timestamp recording to capture of geo-coordinates (Latitude, Longitude).

### Data analysis

Because this was an exploratory study with a small sample size, we calculated frequencies and proportions stratified by subnational and national geographies and facility type for quantitative data. Quantitative data were analyzed descriptively using Tableau Software, version 2023.1.2 (Seattle, USA). Our main analytical focus was on data visualization to compare and contrast results from the nine geographies. This exploratory study was not intended to test a hypothesis. Sample sizes for both country and subnational geographies were purposive and were not calculated to enable statistical comparisons.

Translation and coding of qualitative responses to open-ended questions was conducted using the six-stage scale-up framework as the organizing principle. All data were inductively coded and analyzed using a thematic analysis approach. A team of four coders reviewed and categorized the qualitative data. In specific instances where tie-breaking was necessary, two coders including at least one of the authors (EE, PSC, SK) reviewed and agreed upon data categorization. Excel in Microsoft 365 (Redmond, USA) was used to manage the data.

### Ethical review and approval

The PATH Research Determination Committee reviewed this assessment protocol and determined that it was not human subjects research and did not require ethical review by an IRB. Because of this determination, no other local IRB approvals were sought. This study conformed to the principles embodied in the Declaration of Helsinki and we obtained permissions from the national and subnational Ministry of Health and any other relevant authorities in each country to collect data at each specified health facility. We contacted each health facility prior to our visit to introduce the assessment and coordinate a visit schedule and briefed and debriefed the facility leadership before and after the assessment took place.

We obtained unwitnessed verbal consent from all participants, which was documented in the project database. A consent form describing the assessment procedures, risks, and possible benefits was reviewed with each potential participant prior to beginning the interview. The consent form was read in English or the local language depending on location of the assessment site. All participants were informed that their participation was voluntary and that they could decline to answer any question or participate in any activity that they wish. All stakeholder interviews were conducted in a private setting to ensure confidentiality.

## Results

Between March and October 2021, we worked with Ipsos Group, a multinational market research and consulting firm, to conduct interviews with 275 providers and 94 DHMT staff across nine geographies (Table 2). Given the work was conducted during COVID-19 pandemic with the delta variant outbreak in India in May–June 2021, all possible efforts were made to minimize disruptions to workflows during this process and to pause data collection when the epidemiological picture in the focus geographies warranted.

**Table 2:** Distribution of sites and respondents, by geography.

	Centre Ouest	Sud Ouest	Oromia	Bihar	Uttar Pradesh	Kakamega	Kisumu	Niger	Sokoto	Total
Primary	5	3	10	11	25	2	0	7	10	73
Secondary	5	3	2	13	25	5	8	5	9	75
Tertiary	0	0	1	3	0	2	2	0	1	9
**Total # facilities**	10	6	13	27	50	9	10	12	20	157
DHMTs	5	3	5	12	41	5	5	7	11	94
Providers	20	10	13	48	82	10	10	30	52	275
**Total # informants**	25	13	18	60	123	15	15	37	63	369

### Awareness, experience, and policy translation

**Fig 3:**
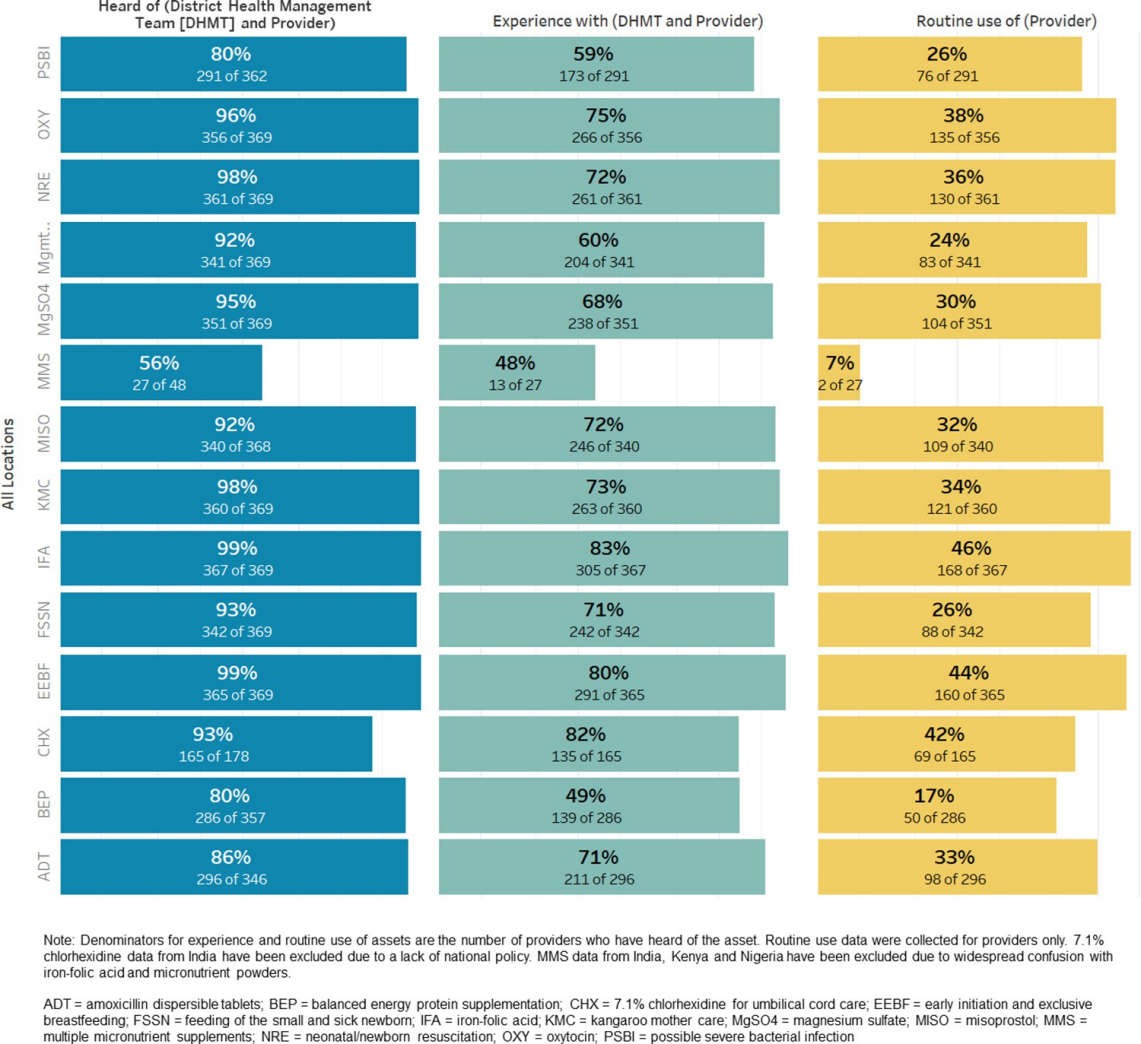
Provider/DHMT awareness, experience, and routine use of assets across geographies. Note: Denominators for experience and routine use of assets are the number of providers who have heard of the asset. Routine use data were collected for providers only. 7.1% chlorhexidine data from India have been excluded due to a lack of national policy. MMS data from India, Kenya and Nigeria have been excluded due to widespread confusion with iron-folic acid and micronutrient powders. ADT = amoxicillin dispersible tablets; BEP = balanced energy protein supplementation; CHX = 7.1% chlorhexidine for umbilical cord care; EEBF = early initiation and exclusive breastfeeding; FSSN = feeding of the small and sick newborn; IFA = iron-folic acid; KMC = kangaroo mother care; MgSO4 = magnesium sulfate; MISO = misoprostol; MMS = multiple micronutrient supplements; NRE = neonatal/newborn resuscitation; OXY = oxytocin; PSBI = possible severe bacterial infection.

Respondents were asked if they had heard of the asset, if they had experience with it, and if they used it routinely. Experience was described as any interaction with the asset. Awareness of most assets was high for DHMT and staff members (Fig 3). At a minimum, most providers and DHMT members had some familiarity with the assets under review. Respondents were less familiar with newer, mostly nutrition assets (e.g., balanced energy protein supplementation, multiple micronutrient supplementation, and PSBI); these were also among the assets with the most limited coverage. More variance was seen in the level of experience in individual assets.

**Fig 4:**
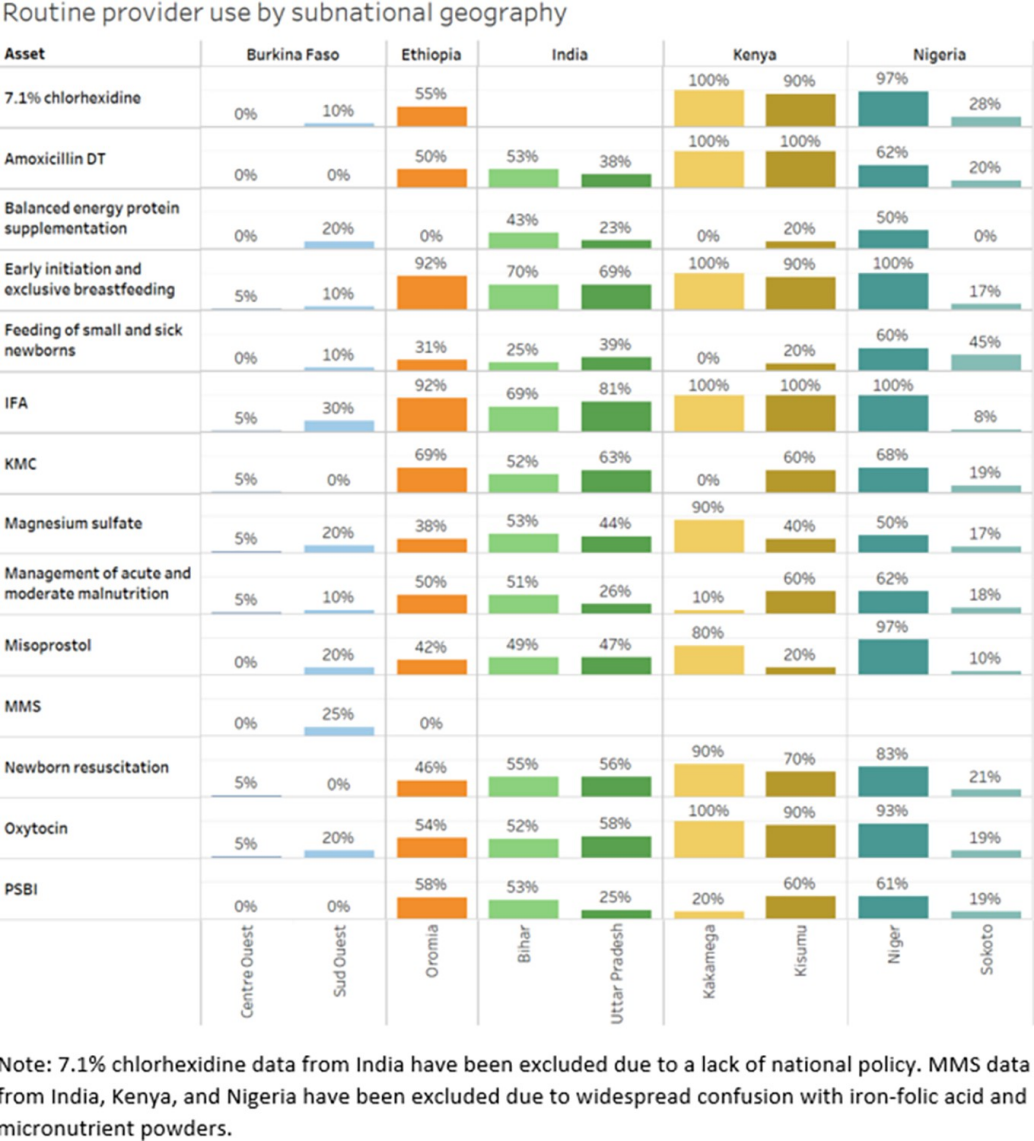
Routine use of select assets as reported by providers, by subnational geography. Note: 7.1% chlorhexidine data from India have been excluded due to a lack of national policy. MMS data from India, Kenya, and Nigeria have been excluded due to widespread confusion with iron-folic acid and micronutrient powders.

Variation exists between and within countries in use of assets (Fig 4). Providers across facilities reported low experience of routine use of certain key facility-based MNCH assets

Translation of policy from national to subnational level might be related to variability in provider use experience. Most respondents (both DHMT and providers) were aware of national policies and were able to provide a summary of clinical guidance associated with each policy. However, most respondents (both DHMT and providers) were either unable to articulate subnational level policies or indicated that they did not exist. Instead, subnational providers articulated clinical guidelines issued by the facility and modeled on national policy and guideline directives. For example, the availability of national guidelines facilitates use and coverage of IFA in Ethiopia:

*Yes, there is a national guideline which highlights the way the IFA is applied. We have also already posted a multiple brochure in Afaan Oromo so that the people of the area can easily understand and apply their activities. Of course, the guideline is written mostly at the national level, but the contextual and locally adopted one also exists and is widely used. The guideline insists us that the IFA service should be given for three months to pregnant women.”*

*–Provider, Oromia, Ethiopia*

Likewise in India, an outlier in successful scale up of KMC, has clear, prominently displayed guidance available for providers at point of service.

*“The guideline highlights that there should be skin-to-skin contact between the mother and child to get the child warm. In cases when a mother is unable to do that, we request one of her relative for the purpose. These types of guidelines exist at our health facility, and we have a poster of it on the wall of the delivery room.”*

*–Provider, Uttar Pradesh, India*

### Health system barriers to asset uptake and use

**Fig 5:**
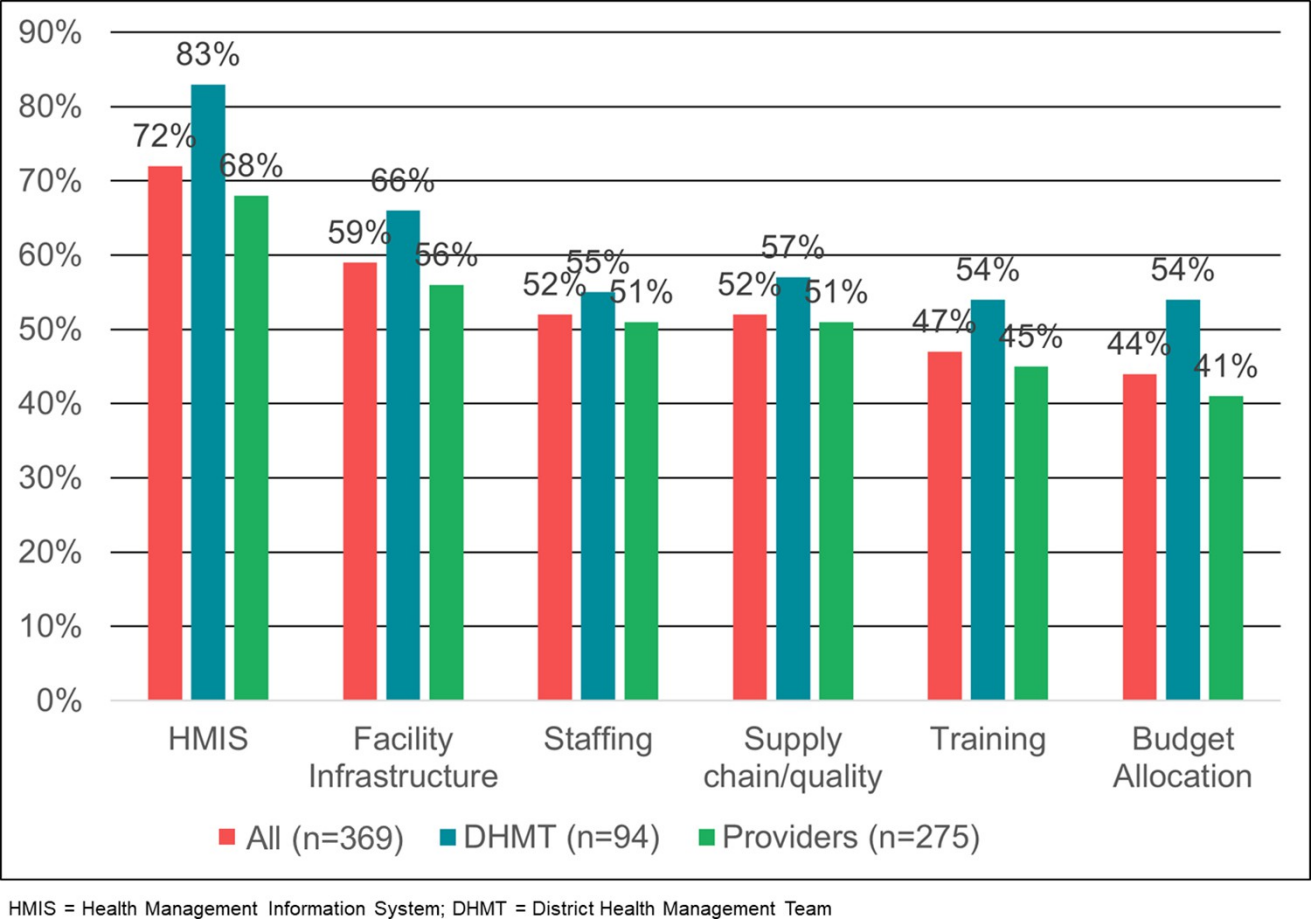
Perception of overall barriers to scale for any asset by respondent category. HIMS = Health Management Information System; DHMT = District Health Management Team.

Provider and DHMT perceptions of barriers to scale for any asset were consistent (Fig 5). HMIS data use was viewed as the largest barrier by any group followed by facility infrastructure. DHMT staff viewed budget allocation and training as a much larger barrier than providers, most likely given their purview as subnational managers. Providers were focused on supply chain and staffing issues which affect workflows and the quality-of-service provision.

**Fig 6:**
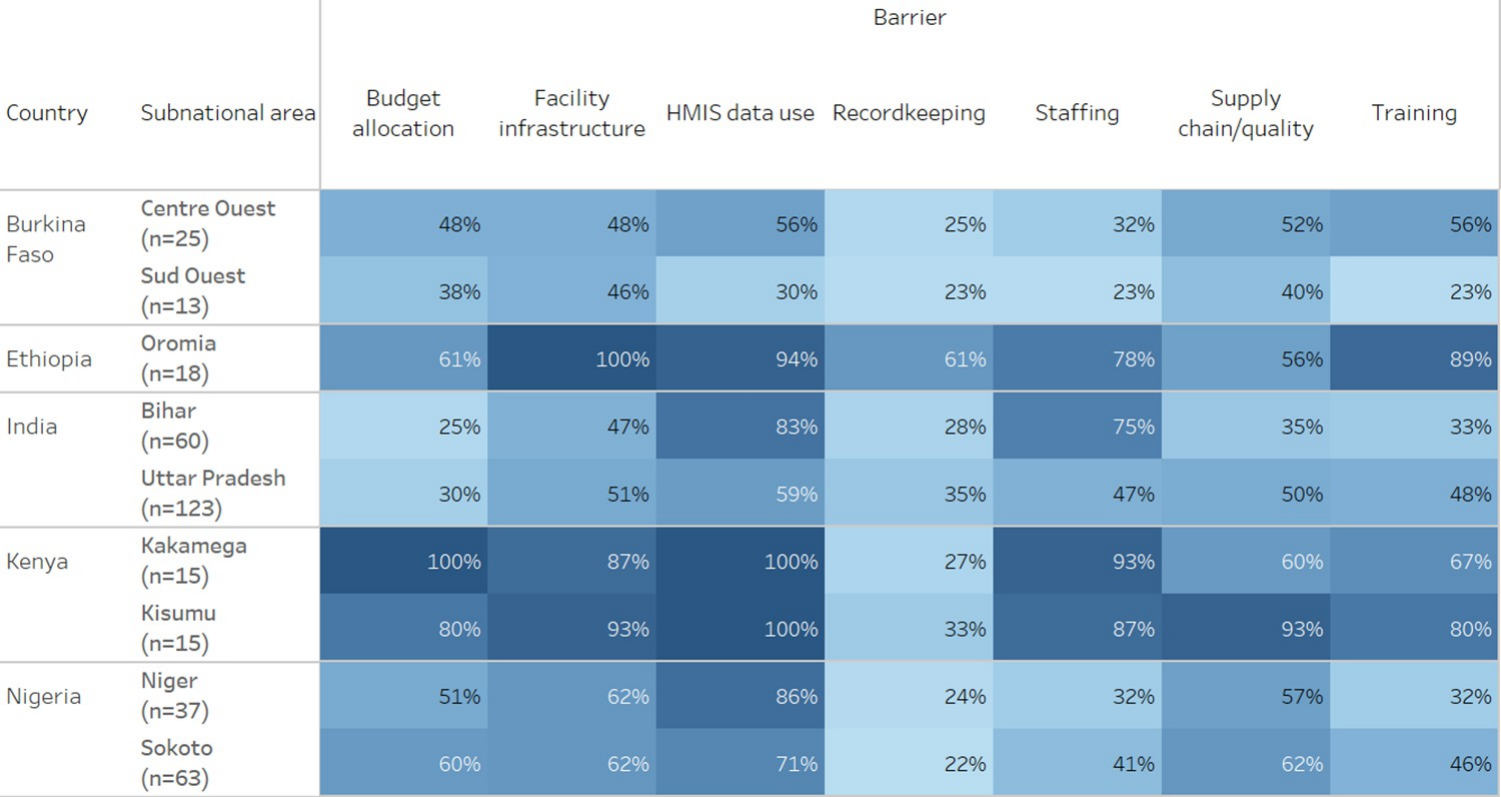
Percentage of DHMT/providers who identified each health system component as a barrier, by geography.

DHMT/provider perceptions of barriers vary by country (Fig 6).

### Data management as barrier

Overall, HMIS data use was identified as the biggest barrier to scale, including 100% of all Kenyan respondents.

*“The biggest barrier can be documentation and that could be due to the staffing when they forget to document.”*

*–Provider, Kakamega county, Kenya*

*“It is not inserted in HMIS, we midwives review by ourself about its progress or when it becomes lowering from the stock but not scheduled review.”*

*–Provider, Oromia, Ethiopia*

Recordkeeping in relation to asset performance was mentioned rarely by DHMT or providers in qualitative data. Assets linked to recordkeeping issues included 7.1% chlorhexidine for umbilical cord care, balanced energy protein supplementation, early and exclusive breastfeeding, management for acute malnutrition, misoprostol, multiple micronutrient supplementation, newborn resuscitation equipment, and oxytocin. The perception of recordkeeping is linked more to physical registers or cards than HMIS systems. Record keeping was not considered to be a key barrier to scale of any asset, regardless of provider type or geography. However, a greater percentage of Ethiopian stakeholders noted recordkeeping as a barrier compared to other geographies:

*“We don’t have record management system.”*

*–Provider, Oromia, Ethiopia*

*“We are not fully implementing it and for other recording we lack basic equipment for documentation.”*

*–DHMT member, Oromia, Ethiopia*

*Recordkeeping was considered a lesser barrier in India and Kenya:*

*“We will not get accurate data. Complete data is not being captured so that is the problem.”*

*–Provider, Uttar Pradesh state, India*

*“Yes, we do the follow up to see whether the data is right or not. Sometimes we mess up because we have loads of reports.”*

*–Provider, Uttar Pradesh, India*

*“We do not know the accuracy because some of us if we write yes in the register, and then if I come and I see them I also write yes. So, am I writing yes because I have done it or is it because I have seen my colleague has done it?”*

*–DHMT, Kisumu county, Kenya*

However, lack of effective recordkeeping systems was called out by stakeholders even in countries where recordkeeping was perceived to be a minimal barrier to scale:

*“Lack of data or proper record keeping…”*

*–Provider, Sokoto state, Nigeria*

*“The absence of a register set up.”*

*–Midwife, Sud-Ouest region, Burkina Faso*

*“We are only written in the registers. No. We have no electricity, no computer.”*

*–Provider, Centre Ouest, Burkina Faso*

### Infrastructure as barrier

Across all countries, infrastructure was mentioned as a barrier by 56% of respondents; DHMT mentioned infrastructure slightly more often than providers (66% vs. 56%). Infrastructure, and particularly the need for electricity, was mentioned in relation to space/use of equipment for small and sick newborn care (i.e., newborn care corner, KMC room, radiant warmers, oxygen). Cold chain facilities were mentioned in the context of oxytocin. Reconfiguring existing spaces to meet use requirements and community ownership around maintenance was mentioned in relation to some specific assets such as KMC and neonatal resuscitation. Strengthening mechanisms for referral to facilities with appropriate and necessary infrastructure might address perceptions of inadequate infrastructure.

All stakeholders in Ethiopia perceived infrastructure to be a barrier to scale:

*“I would say lack of adequate electric power is a problem. By chance, the electricity may not function whenever women are laboring and mostly in the middle of the night or some other random times. In addition, the electricity often does not continuously give service as well. Aside from the electricity related constraints, we rarely face supply-related challenges.”*

*–Provider, Oromia, Ethiopia*

Even in Burkina Faso, where the fewest stakeholders (only about half) perceived infrastructure to be a barrier, concerns were raised:

*“These are the premises, that is, we need a place only for newborns and we need a specialist.”*

*–Midwife, Sud-Ouest region, Burkina Faso*

*“Geographic accessibility of health centres*”

*–Midwife, Sud-Ouest region, Burkina Faso*

### Supply chain and quality of drugs as barrier

Across all countries, supply chain/quality was mentioned as a barrier by 52% of respondents; DHMT mentioned supply chain/quality slightly more often than providers (57% vs. 51%). Supply chain failure impacts affordability of care since patients are required to purchase drugs in the private sector. Better market coordination may help ameliorate stock outs.

In Kenya, supply chain was considered a strong barrier:

*“At times we do not have the drugs, especially some antibiotics we do not have we have to tell them to go and buy and we are not sure if they are buying.”*

*–Provider, Kakamega county, Kenya*

*“The biggest barrier that we have for oxytocin is the supply chain. Sometimes we have the stock out so the county has to come in and look for ways of procuring it.”*

*–DHMT, Kakamega county, Kenya*

On the other hand, in India supply chain was perceived to be less of a barrier:

*“Sometimes availability is a barrier. Sometimes availability is not in a manner that… required quantity is not always available…”*

*–Provider, Uttar Pradesh state, India*

### Staffing as barrier

Across all countries, staffing was mentioned as a barrier by 52% of respondents Staffing shortages, especially of qualified nurses and doctors, were noted; losing qualified staff to shift rotations was also mentioned. In labor and delivery, staff shortages impeded use of assets; a single provider attending both mother and newborn was constrained in effective care provision and use of the full suite of recommended interventions.

Stakeholders in Kenya perceived staffing as a major barrier:

*“Another barrier is the shortage of the healthcare workers, if you have so many deliveries or so many activities, does kangaroo mother care become your priority? So, the shortage is an issue.”*

*–DHMT, Kisumu county, Kenya*

Staffing was identified as a barrier to a lesser extent in Nigeria and Burkina Faso:

*“The biggest barrier is manpower, number of staff, at times when we are doing the resuscitation we are facing the mother at the same time you alone facing the baby it is not supposed to be one person facing the other one, but you will divide yourself, lack of manpower we will be sharing ourself facing the mother at the same time facing the baby, the effective care is supposed to give to both of them will not be there because you are dividing yourself, it is supposed to be 2 people work, that is why I say manpower [sic].”*

*–Provider, Niger state, Nigeria*

*“[Oxytocin was successfully scaled due to] The continuous training of staff, recruited enough staff in health centers, avoided recurrent product breaks and avoid supervision of product stocks.”*

*–Provider, Centre Ouest, Burkina Faso*

### Training as barrier

Training is perceived as initial orientation to a new intervention as well as ongoing supervision and mentoring to assure quality of care. Ethiopian stakeholders identified training as a greater barrier, Nigerian stakeholders less so:

*“There is a skill gap. Firstly, the students are seldom skilled upon their graduation. This is something to do with education quality. Secondly, the previously available specific trainings are missing now. Rather, currently trainings are merged together. For example, in past time there was neonatal resuscitation training. Now, however, it is not available. Not only this, the mentoring and coaching activities are also insufficiently undertaken.”*

*–Provider, Oromia, Ethiopia*

*“The biggest barrier is that this resuscitation is a skill, and anyone who has not acquired the skill will not be able to do it. So, most a times now, they normal recruit health workers that have not had training on that, they don’t anything about newborn resuscitation. So, for all these new health workers who don’t know it, if he is in the labor and a woman is about to deliver, someone has to help him. But thanks be to God there has been a lot of training on that. That everyone should learn how to do resuscitation.”*

*–Provider, Sokoto state, Nigeria*

*“The biggest barrier is lack of training. We don’t have knowledge on it. So, things that you don’t have knowledge on it you cannot implement it. Or you are implementing it wrong.”*

*–Provider, Sokoto state, Nigeria*

### Budget as barrier

Across all countries, budget allocation was mentioned as a barrier by 44% of DHMT/providers; DHMT mentioned budget allocation more often than providers (54% vs. 41%). Budget is directly related to the availability of an asset, which directly impacts quality of care. For DHMT members overall, budget constraints were a concern as it affects all aspects of service delivery: availability of assets, ability to train and mentor staff, and availability of supportive infrastructure. However, there may be self-perceived limitations around how DHMT/providers might influence budget allocation. Budget allocation is perceived as a large barrier by Kenyan stakeholders and much less by Indian stakeholders:

*“The biggest barrier we have is procurement. Sometimes we procure but because of limited funds, we are not able to.”*

*–DHMT, Kakamega county, Kenya*

*“The biggest barrier is from the district level, for us it is available from there, and we make it available at the community level. If we don’t get, then we can’t give also. We have to know what is the barrier at the district level, whether it is budget or it has come to the state level. It is not from the block level, it is at the state or district level, we don’t have fund for it, we cannot purchase it. No barriers from the block level…”*

*–DHMT, Uttar Pradesh, India*

*“In the rural area, biggest barrier is, if it’s not available in hospital and it’s prescribed then they can’t buy it because they belong economic class…”*

*–Provider, Uttar Pradesh, India*

## Discussion

Approaches and models that delineate the process of introduction and scale of evidence-based interventions abound [16–20]. The Pathway to High Effective Coverage is one example of a health systems framework that explicitly incorporates actions at the subnational level throughout five of its six main components (e.g., (1) national readiness; (2) system structures; (3) management capacity; (4) implementation strength; (5) effective coverage; and (6) impact) [21]. Subnational data, such as that collected in this initial exploratory inquiry into how providers/DHMT view the relative order of magnitude of barriers to scale of evidence-based interventions, can be used to inform this type of systems thinking approach.

This assessment offers viewpoints on the process towards scale of MNCHN assets from the distinct viewpoint of providers and DHMT staff. Across all geographies, respondents were less familiar with newer, mostly nutrition, assets (e.g., balanced energy protein supplementation, multiple micronutrient supplementation, and PSBI); these were also among the assets with the most limited coverage.

In the assessment, providers across facilities reported low routine experience use of MNCHN assets. Aggregation across the levels of facilities may have affected routine use (i.e., not all facilities in the sample were designated BEmONC). However, these findings reflect a much lower level of routine use than would be expected given the mix of facilities in the sample. Further, given that limitations in provider skills (i.e., staffing and training) were also identified as a significant barrier to scale, lack of routine use is likely to exacerbate actual and perceived provider efficacy.

Overall, respondents identified data use as the most common barrier. Results from the assessment show that although data collection is uneven, particularly at lower levels of clinical care, data use is valued both from a community and clinical perspective. For example, even in the absence of DHIS2 or HMIS indicators, DHMT/providers reported entering data into HMIS for all 14 assets. This suggests that they perceive “recordkeeping” to be synonymous with HMIS data entry.

We also found that often clinical guidelines at the facility level are called “policies,” so nomenclature for clinical care and public health is not aligned. Per our data, the hypothesis that the translation of a national policy results in a subnational policy does not reflect the situation on the ground.

Many of the barriers to scale identified in our study at subnational level appear to align with barriers identified at national level. For example, results from a multicounty systematic analysis of bottlenecks and proposed solutions to strengthen community health systems undertaken by national level stakeholders in 22 countries in West and Central Africa showed that barriers at the primary health care level related primarily to health financing, essential medical products and technology, and community ownership and partnerships [22]. However, a deep understanding of the contextual characteristics at the subnational level, which can be wholly or partially distinct from the national level, can assist in customizing programmatic approaches that may prove to be successful.

Routine health information systems at subnational level can often be characterized as operating in a constrained environment where there is lack of funding, weak demand to use data, and lack of motivation concerning data quality and use [23]. A recent scoping review on improving quality and use of routine health information system data in low- and middle-income countries categories interventions as i) tools or models facilitating decision-making. ii) technology to improve data quality and data use and iii) capacity building interventions [24], Capacity-building intervention that use data to drive quality improvement at the facility level have been shown to be successful in Africa [25] as well as South Asia [26]. Engaging both health care providers and management staff to prioritize data use may provide an avenue leading to improvements in quality of care. Quality of care improvements will necessarily require addressing other health system gaps such as human workforce training, commodity availability and budget allocation, thus strengthening and enabling the environment for scale.

Over eighty low and middle-income countries have embraced use of the District Health Information System 2 (DHIS2) platform for routine health management of data for an estimated 3.2 billion people [27]. The platform supports both a RMNCAH health data package which generally is used to report on maternal and child mortality in health facilities, infectious disease, antenatal care, and vaccination coverage, excluding most of the assets we investigated in this assessment. The nutrition health data package covers iron-folic acid supplementation and infant and young child feeding only although these indicators may not be implemented universally [28]. Some countries are incorporating additional asset-specific indicators into their updated DHIS2 platform. In Nigeria, two new interventions (amoxicillin dispersible tablets and 7.1% chlorhexidine for umbilical cord care) are included in the most recent DHIS2 update. A recent scoping review [29] about how DHIS2 data are being used for action and decision making found that DHIS2 data is being used but there are few detailed descriptions of this use in peer review or grey literature. It remains to be seen as to whether the addition of new asset-specific indicators into the DHIS2 platform will advance scale of these evidence-based practices.

In this assessment, budget allocation was mentioned as a barrier more often by DHMT staff than providers (54% vs. 41%). At the subnational level, underfunded budgets and misalignment between policy, planning and budgeting sometimes occurs [30, 31]. Results from an analysis of the political economy of subnational health sector planning and budgeting in three counties in Kenya revealed that while devolution has resulted in expanded participation in subnational health management, budget allocation has become more politicized so may not be directly aligned with national priorities [32]. These results align with our findings that one hundred percent of Kenyan DHMT staff respondents identified budget as a barrier to scale for MNCHN assets. In Nigeria, which also uses a decentralized budget allocation process, DHMT staff were much more likely than providers to identify budget as a barrier to scale in both Niger (86% vs. 43%) and Sokoto (73% s. 58%) states. In Oromia, Ethiopia, more DHMT staff (80%) than providers (46%) identified supply chain/product quality as a barrier to scale. In general, less than 60% of providers across all geographies except Kisumu, Kenya (90%) identified supply chain/product quality as a barrier to scale.

Policy translation from national to subnational level can be challenging as evidenced in our assessment where most respondents, regardless of role, were either unable to articulate subnational level policies or indicated that they did not exist. This challenge has been documented elsewhere in Sub-Saharan Africa where new guidelines for postpartum hemorrhage management were not disseminated effectively to frontline workers [33]. This may be related to the influence of social networks maintained by providers and DHMT staff. For example, in Uganda, these networks were shown to act as an enabling mechanism for implementation of specific aspects of policy at the subnational level [34].

This multi-country, subnational investigational approach allowed for assessment of context specific nuances in asset scale-up within and between geographies. A strength of this assessment is that it covers a broad spectrum of maternal, neonatal, child health, and nutrition assets, thus providing a situation analysis across the MNCH spectrum. Additionally, this work captures views of a range of stakeholders delivering services at different levels of facilities.

This assessment was limited in that some respondents were not familiar with newer assets and a sample product for multiple micronutrient supplements, for example, was not available for respondents to see. Also, site selection eligibility required the willingness of district-level authorities to allow data collection at all facilities within the district, which may have added an element of selection bias. Finally, respondent sample sizes in each subnational geography vary widely from a total sample size of 13 in Sud-Ouest to 123 in Uttar Pradesh, a function of the size and an attempt to include multiple types of providers, where significant variation existed. There is also variation by respondent type within these total responses from each geography, ranging from 3 DHMT in Sud Ouest to 41 DHMT in Uttar Pradesh and ten providers in Sud Ouest, Kakamega and Kisumu to 82 providers in Uttar Pradesh. This variation in response by geography may potentially limit drawing generalizable conclusions from our data however given the exploratory nature of our inquiry, we believe that the responses can provide valuable insights into health provider and DHMT perception of barriers to scale that can serve to inform future scale-up and health system strengthening efforts.

Further, review of 14 assets at one time is an enormous undertaking, so the focus was on breadth rather than probes to provide depth of response. Although every effort was made to minimize workflow disruptions, given data collection occurred during the pandemic (and generally before the vaccine was widely available), this may have affected the time stakeholders had available for interviews.

## Conclusion

Health system barriers related to scale vary by geography and type of asset. Understanding perspectives of those most proximate to service delivery (provider and health facility management) of why assets do or do not advance towards effective coverage can assist in creating enabling environments for the scale of interventions. These types of data are perhaps most helpful when collected at the subnational level, which allows for comparisons both within and between countries to show health disparities. Importantly, this strategic data collection can provide a starting point for improvement efforts to address existing health system gaps. In our assessment, both providers and DHMT staff identified HMIS data use as the biggest barrier to scale generally regardless of asset type. This understanding can help guide the trajectory of health system strengthening efforts by identifying an entry point for specific action. This assessment of stakeholder perceptions toward scale is noteworthy because i) data have been collected at a subnational level, where there is a dearth of peer-reviewed reports, ii) the data are able to show health disparities and contextual differences and barriers within and across countries and iii) these results can provide a starting point for overall health systems improvement by identifying the most significant barriers across interventions. At the same time, it also examines unique factors that stakeholders feel have contributed to the scale-up of certain commodities. This provides a unique snapshot of the generic and specific in a single publication which, in our opinion, can also help inform introduction and scale of new MNCH commodities.
